# Supplementary material for: Molecular Geometry Prediction using a Deep Generative Graph Neural Network
Source: Sci Rep. 2019 Dec 31;9:20381. doi: 10.1038/s41598-019-56773-5 (PMC6938476; doi:10.1038/s41598-019-56773-5)
Supplement: Supplementary file 1 — Supplementary Information. [file 41598_2019_56773_MOESM1_ESM.pdf]

Supplementary Information – Molecular Geometry Prediction using a Deep Generative Graph Neural Network

Elman Mansimov[1], **Omar Mahmood**[2], **Seokho Kang**[3], **and Kyunghyun Cho**[1,2,4,5,*]

[1]Department of Computer Science, Courant Institute of Mathematical Sciences, New York University, 60 5th Avenue, New York, New York 10011, United States
[2]Center for Data Science, New York University, 60 5th Avenue, New York, New York 10011, United States
[3]Department of Systems Management Engineering, Sungkyunkwan University, 2066 Seobu-ro, Jangan-gu, Suwon 16419, Republic of Korea
[4]Facebook AI Research, 770 Broadway, New York, New York 10003, United States
[5]CIFAR Azrieli Global Scholar, Canadian Institute for Advanced Research, 661 University Avenue, Toronto, ON M5G 1M1, Canada
[*]Correspondence should be addressed to K.C. (email: kyunghyun.cho@nyu.edu)

ABSTRACT

A molecule's geometry, also known as conformation, is one of a molecule's most important properties, determining the reactions it participates in, the bonds it forms, and the interactions it has with other molecules. Conventional conformation generation methods minimize hand-designed molecular force field energy functions that are often not well correlated with the true energy function of a molecule observed in nature. They generate geometrically diverse sets of conformations, some of which are very similar to the lowest-energy conformations and others of which are very different. In this paper, we propose a conditional deep generative graph neural network that learns an energy function by directly learning to generate molecular conformations that are energetically favorable and more likely to be observed experimentally in data-driven manner. On three large-scale datasets containing small molecules, we show that our method generates a set of conformations that on average is far more likely to be close to the corresponding reference conformations than are those obtained from conventional force field methods. Our method maintains geometrical diversity by generating conformations that are not too similar to each other, and is also computationally faster. We also show that our method can be used to provide initial coordinates for conventional force field methods. On one of the evaluated datasets we show that this combination allows us to combine the best of both methods, yielding generated conformations that are on average close to reference conformations with some very similar to reference conformations.

S1 Hyperparameter Search

Below are the hyperparameters we tried for the QM9 and COD datasets. We picked the hyperparameters to ensure that a model trained with a batch size of 20 molecules could fit on 1 GPU with 12 GB of RAM.

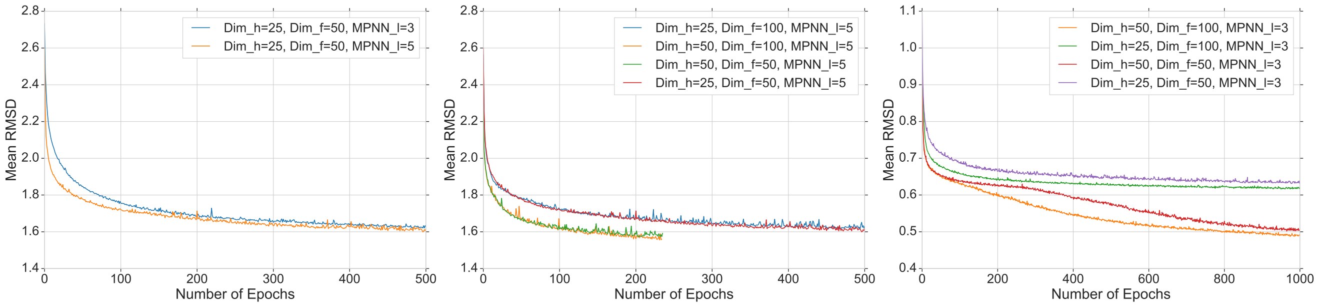

(a) COD dataset **(b)** COD dataset **(c)** QM9 dataset

Figure S1. Investigation of number of different hyperparameters on QM9 and COD datasets over different number of epochs. Mean RMSD over number of epochs of best performing model on valid set of corresponding dataset. Mean RMSD was calculated given 10 conformations per molecule.

Table S1. Node features.

feature	type	dimension
atom type	one-hot (possible heavy atoms)	vary
atomic number	integer	1
chirality	one-hot (R, S)	2
is aromatic	binary	1
hybridization	one-hot (sp, sp^2, sp^3, sp^3d^1, sp^3d^2)	5
degree	integer	1
formal charge	integer	1
no. hydrogens	integer	1
no. radical electrons	integer	1
implicit valence	integer	1
no. rings for each ring size	integer (ring sizes 3, 4, 5, 6, 7, 8)	6
total		> 20

We experimented with the following values of hyperparameters on the QM9 dataset: $d_h = [25, 50]$, $d_f = [50, 100]$. The number of MPNN layers L was fixed to 3 according to previous preliminary experiments. On Figure S1c we can see that the model with $d_h = 50$ and $d_f = 100$ significantly outperforms models with a smaller number of hidden units.

On the COD dataset we experimented with the following values of hyperparameters: $d_h = [25, 50]$, $d_f = [50, 100]$ and number of MPNN layers $L = [3, 5]$. In Figure S1a we can see that the model with 5 MPNN layers slightly outpeforms the model with 3 MPNN layers. Similarly to the QM9 dataset, we can see in Figure S1b that a larger number of hidden units results in significantly faster convergence and better performance.

We selected the model with the best hyperparameter values given by our grid-search. Figure S2 shows the RMSD of this model on the validation set as a function of number of epochs on the QM9 and COD datasets.

S2 Molecular Features

To represent molecules as graph-structured data, each of the nodes and edges in the molecule is represented using the features described in Tables S1 and S2, according to related literature.[1–3] We only consider heavy atoms, and do not consider hydrogen atoms as explicit nodes *i.e.* hydrogen atoms are represented as part of the input features and their coordinates are not predicted by the neural network. In Table S2, the first four edge features are only calculable if the corresponding atom pair is bonded, while the last two edge features are calculable for every atom pair. All features are generated using RDKit.[4]

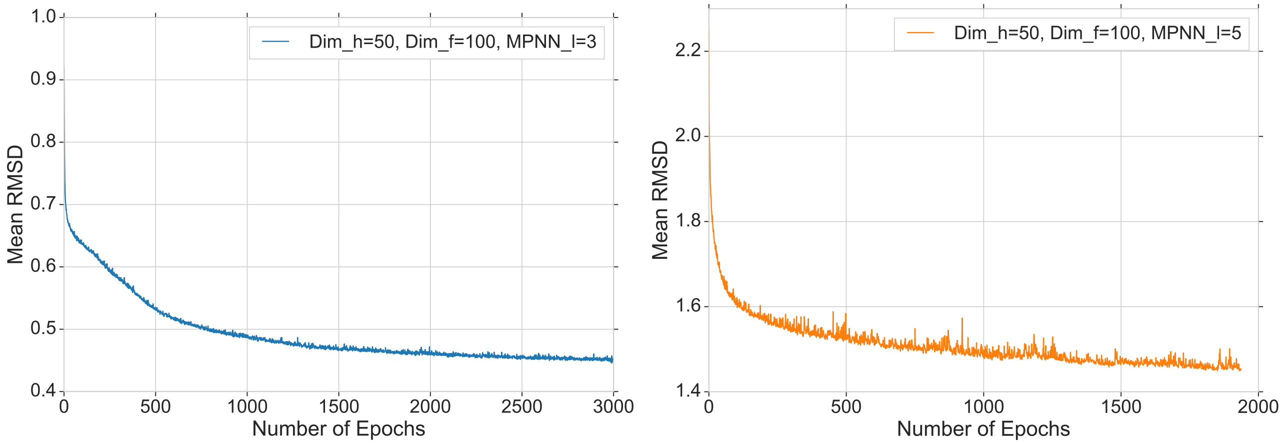

(a) QM9 dataset (b) COD dataset

Figure S2. Performance of the best performing model over the number of epochs. Mean RMSD over number of epochs of best performing model on valid set of corresponding dataset. Mean RMSD was calculated given 10 conformations per molecule.

Table S2. Edge features.

feature	type	dimension
bond type (if bond)	one-hot (single, double, triple, aromatic)	4
stereochemistry (if bond)	one-hot (E, Z)	2
is conjugated (if bond)	binary	1
is in ring (if bond)	binary	1
is in same ring	binary	1
graph distance (shortest path)	integer	1
total		10

References

1. Kearnes, S., McCloskey, K., Berndl, M., Pande, V. & Riley, P. Molecular graph convolutions: Moving beyond fingerprints. *J. Comput. Mol. Des.* **30**, 595–608 (2016).
2. Ramsundar, B., Eastman, P., Leswing, K., Walters, P. & Pande, V. *Deep Learning for the Life Sciences* (O'Reilly Media, 2019).
3. Gilmer, J., Schoenholz, S. S., Riley, P. F., Vinyals, O. & Dahl, G. E. Neural message passing for quantum chemistry. In *Proceedings of the 34th International Conference on Machine Learning*, 1263–1272 (2017).
4. Landrum, G. Rdkit: Open-source cheminformatics. (accessed December 18, 2018).
